# Personality-Related Factors and Depressive Symptomatology Predict Behavioral Control in Patients With Alcohol Use Disorders

**DOI:** 10.3389/fpsyt.2022.866657

**Published:** 2022-07-07

**Authors:** Zofia Lebiecka, Tomasz Skoneczny, Ernest Tyburski, Jerzy Samochowiec, Adam Jędrzejewski, Janina Wirtz, Simone Kühn, Anette Søgard Nielsen, Angelina Isabella Mellentin, Leonie Ascone Michelis, Jolanta Kucharska-Mazur

**Affiliations:** ^1^Department of Psychiatry, Pomeranian Medical University in Szczecin, Szczecin, Poland; ^2^Department of Health Psychology, Pomeranian Medical University in Szczecin, Szczecin, Poland; ^3^Neuroplasticity Research Group, Department of Psychiatry and Psychotherapy, University Medical Center Hamburg-Eppendorf, Hamburg, Germany; ^4^Unit for Clinical Alcohol Research, Unit for Psychiatric Research, Department of Clinical Research, Odense Center, University of Southern Denmark, Odense, Denmark

**Keywords:** alcohol use disorders (AUDs), personality, depression, behavioral control, impulsivity, response inhibition

## Abstract

In the face of increasing social, economic, and health consequences of alcohol use disorders (AUDs) and limited effects of available treatment options, the search for novel prevention and management methods continues to remain a timely and valid endeavor. This, however, requires a better grasp of the theoretical framework underlying addiction mechanisms. With the goal to extend the existing body of evidence on AUDs, we set out to investigate the effect of personality-related factors and depressive symptomatology on (i) impulsivity, (ii) cognitive response inhibition, and (iii) the links between the two measures of behavioral control (different facets of impulsivity and response inhibition) in a treatment-seeking AUD sample. To this end, 53 male (*n* = 45) and female (*n* = 8) inpatients at an alcohol rehabilitation center completed three self-report questionnaires: the International Personality Item Pool (IPIP-50), the Beck Depression Inventory Second Edition (BDI-II) and the Barratt Impulsiveness Scale (BIS-11) and performed one behavioral task—an alcohol go/no go task. Regression analyses revealed conscientiousness, intellect, and depression level to be important potential predictors of self-report impulsivity and processing speed in recovering drinkers. No significant links were observed between the two measures of behavioral control, thus complementing evidence that while they both encompass behavioral under-regulation, they may indeed represent distinct psychological constructs.

## Introduction

As the addiction-related adverse public health consequences, and respective social and economic burden to both individuals and societies worldwide are growing, so is the importance of more efficaciously addressing the issue of substance use disorders (SUDs). Despite all the existing treatment options, SUDs remain difficult to manage, with relapse rates reaching approximately 50% across different populations (1). Among all addictions, an especially harmful one, and thus a major health concern is that to alcohol, contributing to over 200 disease and injury conditions. Furthermore, alcohol use disorder (AUD) is responsible for about 3 million deaths annually, while taking a particular toll on young individuals between 20 and 39 years of age (2).

Even though the theoretical underpinnings of alcohol use disorders (AUDs) seem to be quite well-established, available relapse prevention and treatment methods remain insufficient. This is to say that while there exist a number of efficacious evidence-based treatments for AUD, they do not seem to be successful for all patients (3). This tentatively implies that theoretical frameworks concerning underlying addiction mechanisms may still need to be further refined and expanded—with a hope to improve the contemporary clinical paradigms. Albeit these already combine the use of medication and cognitive–behavioral interventions, further amplification of the effects by broadening the existing assessment of related mechanisms and thus treatment options, is warranted c.f. (4, 5).

AUD has been conceptualized in terms of a certain imbalance between impulsive and reflective systems (6), whose ICD-10 criteria include an irresistible desire or compulsion to use alcohol (i.e., craving/limbic adaptation), difficulties to control its intake (i.e., compulsion/lack of impulse control), evidence of physiological withdrawal and tolerance (i.e., physiological adaptation), gradual neglect of alternative pleasures/interests (i.e., narrowed attention/attentional bias), and persistent use despite harmful consequences (i.e., lack of conscious control despite insight) (7). Of note, craving constitutes not only a fundamental diagnostic criterion, but also a significant relapse predictor (8, 9), and therefore is worthy of further study with regard to assessment and treatment models.

Like its predecessor, also the eleventh revision of the International Classification of Diseases and Related Health Problems (ICD-11) considers alcohol use disorder to be the central diagnostic entity concerning its pathological use, whose key feature is deemed a strong internal drive to drink linked to impaired control, typically (but not necessarily) accompanied by a feeling of craving (10). Though seemingly only minor, there are, indeed, changes in the approach toward AUDs in ICD-11 relative to ICD-10, which may not only dictate the use of different and hopefully more efficacious management paradigms in the future, but which, more importantly, suggest that despite numerous studies to date, there is still more to discover as far as the fundamentals of AUDs are concerned.

It is quite commonplace to couple addictive disorders with an entire array of psychological and social factors considered likely contributors to their development and maintenance, with personality characteristics as most promising candidates underlying alcohol misuse (11, 12). Most empirical focus seems to have been centered around the Big Five personality traits, with research demonstrating how each of its domains (neuroticism, extraversion, openness to experience, agreeableness, and conscientiousness) is associated with alcohol use (13). Nevertheless, continuous endeavors to establish the addictive personality profile have been yielding diverse results, suggesting that each addiction, though sharing certain common key features (cf. addiction diagnostic guidelines), may, in fact, be linked with and reflect a distinctive underlying personality and its development. Personality traits have been conceptualized not only to constitute genetic phenotypes disposing for alcohol use (14), but also driving forces behind the motivations to engage in drinking behaviors as well as behind the very drinking paradigms (15). Hence, individually targeted interventions have proven useful in prevention of AUDs and other addictions (16, 17).

Even though links between personality and alcohol use have been investigated across diverse populations (18–22) research has yielded mixed results, suggesting their varied etiology. The factors considered central to the Big Five model are postulated to include such descriptive characteristics as: excitable and easily upset for neuroticism; talkative, assertive, energetic for extraversion; intellectual, imaginative, independent-minded for openness; good-natured, cooperative, trustful for agreeableness; and orderly, responsible, dependable for conscientiousness (13). And so, the most widely studied: neuroticism and related constructs have been associated with coping-motivated alcohol use, while those linked to extraversion have been reported as accountable for social drinking (23, 24). Though there are findings attributing alcohol misuse to neuroticism, triggering pathological coping mechanisms in response to the underlying negative emotionality [e.g., (25)], further evidence is lacking to support such observations (26, 27). As for the other Big Five traits, lower conscientiousness and lower agreeableness were reported to be associated with greater alcohol misuse *via* a more antisocial paradigm of alcohol consumption and lesser inclination to assume adult roles as well as responsible manner of conduct [e.g., (28)]. In two different studies, Zilberman et al. (29, 30) found all addiction populations to manifest greater impulsivity and neuroticism relative to controls, while individuals with AUDs to also score lower on extraversion, agreeableness, and openness to experience. Next to low agreeableness (particularly the facets compliance and straightforwardness), meta-analytic evidence seems to implicate also lower conscientiousness (mostly the facets deliberation and dutifulness) as a likely predictor of pathological alcohol consumption, with certain facets of extraversion (i.e., excitement seeking) and neuroticism (i.e., impulsiveness and angry hostility) considered accountable for affecting drinking behaviors and alcohol-related problems (15). What this brief review of evidence therefore suggests is that rather than factors *per se*, it may be certain profiles of characteristics that seem to be related to AUDs.

Nevertheless, despite the quite abundant body of evidence concerning AUD, the question still remains whether it may, in fact, be a product of a more complex interplay among various psychosocial factors, with growing evidence implicating impulsivity as one of its central determinants. A neurocognitive perspective dictates that human capacity to resist craving and make adaptive decisions is regulated by the reflective system, involving i.a. the mechanisms of cognitive inhibitory control and delayed gratification. Conversely, the reflexive processes commonly linked to impulsive and risky behaviors are associated with the automatic (primarily emotional) responses to reward, rendering faster approach tendencies (31). Quite notably, as a multi-faceted notion, impulsivity involves both (impulsive, affect-dominated) choice (when a smaller, but more prompt reward is selected over a delayed one) and (impulsive) action (originating in one’s incapacity to inhibit a dominant behavior) (32), attributable to the underlying reward-seeking mechanisms or poor inhibitory control. It is, thus, deficits and perturbations within both reflective and reflexive systems that are postulated to be accountable for the combination of compulsive substance seeking and reduced controlled decision-making ability observed in AUDs. And so, there is evidence of impulsivity’s effect on alcohol use outcomes (33) and the severity of alcohol dependence (34). Patients with AUDs have also been reported to manifest cognitive deficits, with high impulsivity scores among them (35, 36) and impaired decision-making due to elevated impulsivity (37). Nevertheless, other findings suggest that heavy alcohol use may be linked with different facets of impulsivity (as it is in the case of personality-related factors), which may motivate its consumption in numerous ways, suggesting there is more area to investigate, thus implicating, e.g., the deficient response inhibition that seems to be a risk factor here due to its association with increased craving in response to alcohol cues (38).

Not surprisingly then, a number of social and environmental cues (such as substance-related settings, interactions, and paraphernalia) combined with individual sensitivity may be implicated in the development of AUDs on the one hand and maintenance of abstinence on the other (39). Of note, vulnerability to alcohol-related cues accompanied with reward anticipation are likely triggers of, first, a neural (reward pathway activation), and second, a subsequent behavioral response. Available neurobiological evidence c.f. (40–42) suggests that SUDs (AUDs included) may stem from impaired neuroplasticity and that the presence of substance-related socio-environmental cues may elicit behavioral and corresponding neurobiological responses, thus linking impulsivity, social cues, and cognitive function. Quite notably, compared to light drinkers, alcohol-dependent drinkers exhibit stronger reactions to alcohol-related cues and personality factors may be involved in cue reactivity, with impulsivity a likely candidate (38).

Another significant marker of AUD, not to mention a likely target of intervention is impaired control over drinking (43, 44). Though related to impulsivity, it is still a conceptually distinct notion (45, 46), the former constituting a so-to-speak behavioral trigger, while the latter is understood in terms of a response inhibition tool. Transition to AUD is postulated to involve a shift from impulsive toward compulsive behavior, which entails impairment within executive control processes (47), i.e., a potential facet of a more general breakdown in behavioral control (understood in terms of the ability to activate and inhibit behavioral responses) (48). Interestingly, the role of impaired control over alcohol consumption in its associations with impulsivity is less clear and requires further investigation (49).

In addition, it remains unknown whether the postulated relationships between personality, impulsivity and impaired control over alcohol use in AUD may be in any way affected by comorbid depressive disorder, which remains a research priority given the high incidence of mood dysregulation in clinical samples (49). Depression is frequently associated with at-risk drinking c.f. (50), both highly prevalent, with approximately 280 million adults affected (51), 20% of whom also reported to meet diagnostic criteria for AUD (52). There is a theorized role of negative emotional state underlying addiction pathology (53), and links between depressive symptoms and failures in drinking control have been postulated in literature (44). In view of the emergence of the COVID-19 pandemic, whose aftermath has contributed to exacerbation of numerous determinants of poor mental health (54), including depression and substance use disorders, the urgency to target the links between them seems greater than ever.

Given the detrimental impact of compulsive alcohol consumption on various aspects of human functioning and the still insufficient methods to combat AUDs, the pursuit of their likely determinants remains timely and valid. In view of that, in this study we sought to investigate the effect of personality-related factors and depression symptomatology level on (i) impulsivity, (ii) cognitive response inhibition, and (iii) the links between the two measures of behavioral control (different facets of impulsivity and response inhibition) in a treatment-seeking AUD population.

## Materials and Methods

### Participants

Fifty-three Polish male (*n* = 45) and female (*n* = 8) inpatients of an alcohol rehabilitation center, aged 25–62 years (*M* = 42; *SD* = 8.94) were recruited to participate in an international study (for more information see) (55) evaluating the effect of modern technologies on enhancing treatment as usual (TAU) in the therapy of AUDs. As part of this larger project, upon screening for eligibility and undergoing a baseline interview, all participants were asked to complete a questionnaire set, including the International Personality Item Pool (IPIP-50), the Beck Depression Inventory Second Edition (BDI-II) and the Barratt Impulsiveness Scale (BIS-11), and to perform an alcohol go/no go task. The assessment was carried out within 2 weeks from admission.

TAU lasted for approximately 2 months and consisted mainly of psychological CBT interventions, applied during individual and group sessions. The treatment incorporated psycho-education, functional analysis of drinking situations, development of coping strategies, problem-solving, and homework between the sessions. Prior to enrollment, each patient was provided with written and oral information about the project and gave their formal consent to participate.

Eligibility criteria included: (1) written informed consent to participate in the study; (2) age ≥ 18 years; (3) completed detoxification (if needed); (4) no sensory or motor deficits complicating administration of the alcohol go/no go task; (5) no other SUDs; and (6) no severe psychiatric or neurological comorbidity or terminal somatic illness.

Baseline assessment was carried out with the use of the Mini-International Neuro-psychiatric Interview (MINI) for DSM-5, a structured interview probing the 17 most prevalent psychiatric diagnoses *via* a set of dichotomous yes/no questions (56, 57).

### Psychological Assessment

Personality assessment was performed with the use of the 50-item International Personality Item Pool—a self-report personality test developed by Goldberg (58) to measure the Big Five personality traits, as expressed in Costa and McCrae’s (59) revised NEO personality inventory (NEO-PI-R). The tool is reported to correlate with the NEO-PI-R domain scores ranging between 0.85 and 0.92. Interestingly, the IPIP-50 scales were also reported to outperform their NEO-PI-R counterparts as predictors of various clusters of self-reported behavioral acts.

Depressive symptomatology was measured with the Beck Depression Inventory Second Edition (BDI-II), a 21-item self-report questionnaire and one of the most widely applied psychometric tools for assessing the severity of depression. The test has good reliability (Pearson *r* = 0.93) and internal consistency values (α = 0.91) (60).

The 30-item Barratt Impulsiveness Scale (BIS-11) was used to assess three facets of impulsivity: (1) attentional (attention and cognitive instability), (2) motor (motor and perseverance); and (3) non-planning (self-control and cognitive complexity). Responses were recorded on a 4-point Likert scale, ranging from 1 = very true for me to 4 = very false for me (61).

Behavioral control was tested with a modified version of the classical Go/No-Go Task [for a detailed description see (56)], typically applied to assess response inhibition (62, 63). The modification involved the use of a set of alcohol-related and neutral visual content to test for inhibition capacity toward alcohol-related cues. Patients were instructed to respond as fast as possible, and without errors to pictures of alcoholic and non-alcoholic drinks appearing on a computer screen, by pressing the space button in response to a non-alcoholic one (i.e., “Go” signals) and to withhold their response when they saw an alcoholic drink (i.e., “NoGo” signals). The two tested measures were response time to “Go” signals as the measure of processing speed, and number of errors as the indicator of capacity to inhibit prepotent response.

### Statistical Analysis

All statistical analyses were performed using SPSS 27 and AMOS 7. The normality of distributions was checked using the Shapiro-Wilk test, kurtosis, and skewness. Skewness and kurtosis between –2 and + 2 were assumed to indicate normal distribution of variables (64). Bivariate relationships were assessed with the Pearson *r* coefficients. Furthermore, Structural Equation Model (SEM) procedure was used to investigate the impact (multiple regression model) of personality and depressive symptomatology on impulsivity and processing speed. The selected indices were: the chi-square statistic *(x^2^)*, the root mean square error of approximation (RMSEA) (65), the goodness-of-fit index (GFI) (66), and the comparative fit index (CFI) (67). The RMSEA of < 0.06, 0.08–0.10, and > 0.10 were considered to indicate good, adequate, and poor scores, respectively, and GFI, and CFI of > 0.90 were considered to indicate an acceptable fit (68). We used a bootstrap maximum-likelihood estimation with 2,000 samples. Additionally, for a more in-depth investigation of differences between facets of personality and impulsivity, repeated measures analysis of variance (RM-ANOVA) was performed. We used pairwise comparison with Bonferroni and Greenhouse-Geisser corrections for degrees of freedom (only for personality, as Mauchly’s test of sphericity proved significant). For impulsivity, the three facet scores were transformed into unitarized units using the formula x_*u*_ = [(x_*i*_–min)/(max–min) × 100] (ranges from 0 to 100, the higher the score, the greater the self-reported impulsivity). The transformation was necessary as the attentional scale had 8 items only (i.e., fewer than the other two, each one including 11 items).

## Results

### Participant Characteristics

The sample consisted of 53 male and female participants (*n* = 45 and *n* = 8, respectively), aged 25–62 years (*M* = 42; *SD* = 8.94), reporting between 8 and 26 years of education (*M* = 13.377; *SD* = 2.989). Drinking initiation age (concerning alcohol intake in any amount at least 3 times per week) ranged between 13 and 51 years (*M* = 25.923; *SD* = 9.416), while duration of drinking (≥ 3 times per week) ranged between 1 and 46 years (*M* = 17.154; *SD* = 9.950). In the last 30 days prior to the study, the participants reported between 0 and 20 days of alcohol consumption (*M* = 5.358; *SD* = 5.582).

As for the assessed personality-related factors, the participants scored highest on agreeableness (*M* = 34.604; *SD* = 5.333), followed by intellect (*M* = 32.925; *SD* = 3.413), conscientiousness (*M* = 32.264; *SD* = 6.355), emotional stability (*M* = 30.302; *SD* = 4.263), and extraversion (*M* = 24.906; *SD* = 6.307). The observed differences were significant, [*F*_(2.84, 147.43)_ = 25.51; *p* < 0.001; ɳ^2^ = 0.33], occurring between extraversion and all other traits (*p* < 0.001), agreeableness and emotional stability (*p* = 0.001), and intellect and emotional stability (*p* = 0.005). Depression symptom severity across the sample reached the mean of *M* = 11.434 (*SD* = 8.520), corresponding to minimal severity. Of the three investigated facets of self-report impulsivity, the patients scored highest on non-planning (*M* = 14.887; *SD* = 5.243), followed by motor (*M* = 10.132; *SD* = 4.532) and attentional impulsivity (*M* = 8.075; *SD* = 3.310). The observed significant differences [*F*_(2, 104)_ = 21.71; *p* < 0.001; ɳ^2^ = 0.30] between attentional impulsivity and non-planning (*p* < 0.001), and motor impulsivity and non-planning (*p* < 0.001), suggest a significantly higher inclination toward non-planning relative to the two other aspects of impulsivity in our sample. Mean response time (in ms) to Go trials in the Go/No Go task equaled *M* = 607.421 (*SD* = 86.844), while mean number of errors was *M* = 1.057 (*SD* = 1.499).

Detailed demographic, clinical, and psychological participant characteristics are presented in Table 1.

**TABLE 1:** Demographic, clinical, and psychological characteristics of participants.

	*M*	*SD*	Min	Max
**Demographic and clinical variables**				
Age	42.000	8.940	25	62
Years of education	13.377	2.989	8	26
Drinking initiation	25.923	9.416	13	51
Duration of drinking	17.154	9.950	1	46
Days of drinking for 30 days	5.358	5.582	0	20
Drinking initiation with intoxication	28.923	11.784	0	51
Duration of drinking with intoxication	9.385	8.388	0	44
Drinking with intoxication for 30 days	3.245	4.751	0	20
Abstinence	0.298	0.462	0	1
**Psychological variables**				
Extraversion in IPIP-50	24.906	6.307	15	42
Agreeableness in IPIP-50	34.604	5.333	24	47
Conscientiousness in IPIP-50	32.264	6.355	20	49
Emotional stability in IPIP-50	30.302	4.263	18	42
Intellect in IPIP-50	32.925	3.413	25	40
Depression symptomatology in BDI-II	11.434	8.520	0	35
Attentional impulsivity in BIS-11	8.075	3.310	3	18
Motor impulsivity in BIS-11	10.132	4.532	3	21
Non-planning impulsivity in BIS-11	14.887	5.243	2	26
Reaction time in GNG	607.421	86.844	466	824
Errors in GNG	1.057	1.499	0	7

*BDI-II, Beck Depression Inventory Second Edition; BIS-11, Barratt Impulsiveness Scale; GNG, Go/No-Go Task; IPIP-50, International Personality Item Pool.*

### Personality, Depression Symptomatology, and Self-Report Impulsivity

Table 2 presents correlation coefficients for the relation of personality traits, depressive symptoms and self-report impulsivity. Not all personality traits were found to correlated with impulsivity. Extraversion was significantly related to attentional impulsivity (*r* = 0.379; *p* < 0.01) and non-planning (*r* = 0.356; *p* < 0.01). In turn, significant negative relationships were observed between agreeableness and non-planning (*r* = –0.349; *p* < 0.05), and between intellect, motor impulsivity (*r* = –0.304; *p* < 0.05) and, again, non-planning (*r* = 0.484; *p* < 0.001). Interestingly, depression symptomatology was significantly positively related to all three facets of impulsivity, i.e., attentional (*r* = 0.529; *p* < 0.001), motor (*r* = 0.370; *p* < 0.01), and non-planning (*r* = 0.340; *p* < 0.05).

**TABLE 2:** Pearson correlation coefficients for the relation of personality traits and depression symptomatology with impulsivity.

	Attentional impulsivity in BIS-11	Motor impulsivity in BIS-11	Non-planning impulsivity in BIS-11
Extraversion in IPIP-50	0.379**	0.226	0.356**
Agreeableness in IPIP-50	–0.124	0.050	−0.349*
Conscientiousness in IPIP-50	–0.244	–0.081	−0.610**
Emotional stability in IPIP-50	–0.188	–0.193	0.039
Intellect in IPIP-50	–0.180	−0.304*	−0.484***
Depression symptomatology in BDI-II	0.529***	0.370**	0.340*

BDI-II, Beck Depression Inventory Second Edition; BIS-11, Barratt Impulsiveness Scale; IPIP-50, International Personality Item Pool. *p < 0.05. **p < 0.01. ***p < 0.001.

#### Personality, Depression Symptomatology, and Behavioral Control

Table 3 shows the correlation coefficients for the relation of personality traits and depression symptomatology with behavioral control. The only significant correlation was the negative one observed between intellect and response time as a measure of processing speed (*r* = –0.344; *p* < 0.05).

**TABLE 3:** Pearson correlation coefficients for the relation of personality traits and depression symptomatology with cognitive response inhibition.

	Reaction time in GNG	Errors in GNG
Extraversion in IPIP-50	0.149	0.058
Agreeableness in IPIP-50	0.093	–0.038
Conscientiousness in IPIP-50	0.156	–0.038
Emotional stability in IPIP-50	0.136	0.051
Intellect in IPIP-50	−0.344*	0.008
Depression symptomatology in BDI-II	0.00	–0.125

BDI-II, Beck Depression Inventory Second Edition; GNG, Go/No-Go Task; IPIP-50, International Personality Item Pool. *p < 0.05.

#### Impulsivity and Cognitive Response Inhibition

Correlation coefficients for self-report impulsivity and cognitive response inhibition are presented in Table 4. No significant links were found between the two measures of behavioral control.

**TABLE 4:** Pearson correlation coefficients for impulsivity and cognitive response inhibition.

	Reaction time in GNG	Errors in GNG
Attentional impulsivity in BIS-11	0.089	–0.094
Motor impulsivity in BIS-11	0.174	–0.015
Non-planning impulsivity in BIS-11	0.103	0.047

*BIS-11, Barratt Impulsiveness Scale; GNG, Go/No-Go Task.*

#### Personality and Depression Symptomatology as Predictors of Self-Report Impulsivity and Processing Speed

**FIGURE 1:**
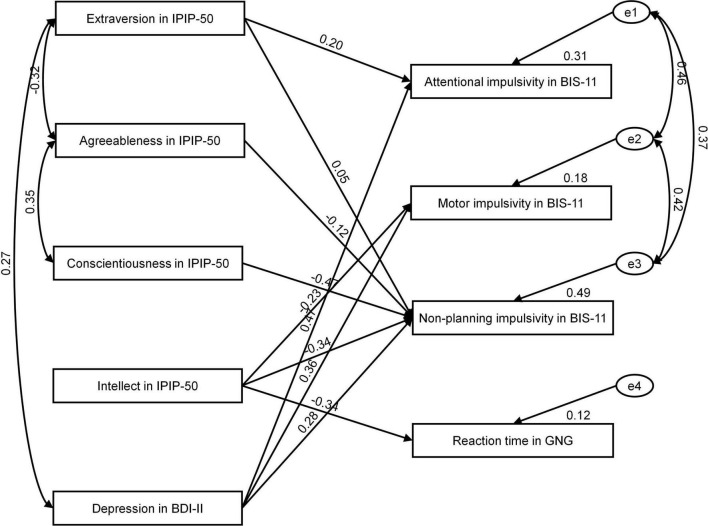
Personality and depression symptomatology as predictors of impulsivity and processing speed.

We adopted path analysis methodology within a Structural Equation Model (SEM) framework to test the effect of personality and depression symptomatology on impulsivity and behavioral control. Considering only significant correlations, we decided to add to the model selected paths between personality, depression symptomatology level, and measures of impulsivity and processing speed. Based on the criteria recommended by Hu and Bentler (68), the model showed good fit to data (χ^2^ = 21.99 and *p* = 0.341; RMSEA = 0.044 and *p* = 0.493; GFI = 0.916; CFI = 0.983) (see Figure 1).

Table 5 presents standardized regression weights for the effects of the four personality dimensions and depression symptomatology on impulsivity and processing speed. As shown, conscientiousness and intellect had an overall effect on non-planning (β =-0.471; *p* < 0.01 and (β = –0.339; *p* < 0.01, respectively). In addition, intellect had an effect on reaction time (β = –0.344; *p* < 0.05). Interestingly, depression symptomatology had an effect on all three aspects of impulsivity [attentional (β = 0.471; *p* < 0.01), motor (β = 0.357; *p* < 0.05), and non-planning (β = 0.278; *p* < 0.05)]. Recorded values of predicting variance were 12% for processing speed, 18% for motor impulsivity, 49% for non-planning impulsivity, and 31% for attentional impulsivity. In general, conscientiousness, intellect, and depression symptomatology were important predictors of self-report impulsivity and processing speed. That is, patients who scored higher on conscientiousness and intellect were likely to describe themselves as less impulsive but tended to respond faster. Moreover, participants with higher levels of depression symptomatology tended to have poorer overall control of impulsivity.

**TABLE 5:** Standardized regression weights for all relations.

	Estimate	Lower	Upper
Extraversion in IPIP-50—Attentional impulsivity in BIS-11	0.196	–0.054	0.422
Extraversion in IPIP-50—Non-planning impulsivity in BIS-11	0.054	–0.149	0.290
Agreeableness in IPIP-50—Non-planning impulsivity in BIS-11	–0.124	–0.361	0.141
Conscientiousness in IPIP-50—Non-planning impulsivity in BIS-11	−0.471**	–0.687	–0.240
Intellect in IPIP-50—Motor impulsivity in BIS-11	–0.225	–0.469	0.062
Intellect in IPIP-50—Non-planning impulsivity in BIS-11	−0.339**	–0.501	–0.099
Intellect in IPIP-50—Reaction time in GNG	−0.344*	–0.580	–0.063
Depression symptomatology in BDI-II—Attentional impulsivity in BIS-11	0.471**	0.220	0.660
–Depression symptomatology in BDI-II—Motor impulsivity in BIS-11	0.357*	0.055	0.606
Depression symptomatology in BDI-II—Non-planning impulsivity in BIS-11	0.278*	0.013	0.504

BDI-II, Beck Depression Inventory Second Edition; BIS-11, Barratt Impulsiveness Scale; GNG, Go/No-Go Task; IPIP-50, International Personality Item Pool. *p < 0.05. **p < 0.01.

In addition, we checked the relationship between age and other psychological variables. Only one significant positive correlation emerged between age and extraversion (*r* = 0.27; *p* = 0.048). As it was not related to the dependent variables, we did not add it to the SEM model.

## Discussion

In this study we demonstrate the effect of personality-related factors and depression symptomatology level on self-report impulsivity and processing speed as the two measures of behavioral control in a treatment-seeking AUD population.

Quite surprisingly, of the five tested personality-related factors, our sample scored the highest on agreeableness and intellect (equivalent to openness), which is quite contrary to the findings of other authors, who tend to report their low levels in AUD cohorts [cf. (29, 30, 15)]. In turn, lower extraversion and emotional stability corresponding to higher neuroticism seem to remain much in line with the results cited in other findings. This particular personality profile, where agreeableness is unexpectedly the most prominent trait, may reflect the fact that our sample was composed of treatment-seeking rehabilitation center inpatients, whose willingness and readiness to conform to certain rules is, by definition, greater than average or otherwise they would not be able to meet admission requirements (a voluntary seclusion in a constrained environment). In turn, intellect, equivalent to openness to experience, may assume predominant values, corresponding with their general readiness to challenge convention, traditional rules, or authority and seek extra stimulation, which is in accordance with descriptions of psychological mechanisms underlying addictive behaviors (69).

According to our findings, certain personality domains and depression symptomatology may predict self-report impulsivity and processing speed, considered two facets of behavioral control in AUD patients. Deemed central to addiction, impaired control could be construed as a multidimensional notion, encompassing impulsivity with its internal latent structure postulated in literature [e.g., (70)]. In the current study, we focused on what could be broadly labeled as impulsive personality trait, i.e., self-reported self-regulatory capacity, and impulsive action, i.e., the capacity to inhibit a prepotent motor response (to alcohol stimuli), which is reflected by: (i) the number of errors and (ii) response time, i.e., processing speed in the Go/NoGo task, and their associations with personality-related factors and self-reported depression symptomatology.

As for the former, in our sample, conscientiousness and intellect seemed not only to correlate with, but also have an overall effect on one of the facets of impulsivity that is non-planning. Our results therefore suggest that greater intensity of (either of) the two traits makes individuals with AUD more likely to engage in future planning and forethought about consequences of their actions, which could also contribute to greater control over alcohol-related consequences. Incidentally, non-planning has been found to be associated with alcohol-related outcomes (71). Of note, although most prevalent across our sample, agreeableness has been deemed insignificant in the applied SEM model.

As for the latter, although deficits in prepotent response inhibition have been associated with diminished capacity to control substance use (72), also in individuals with alcohol dependence (73), in this study we sought to investigate the mechanisms underlying this phenomenon. We found intellect to have an effect on reaction time (c.f. its negative correlation with the Go/NoGo task considered a measure of processing speed). This means that patients who scored higher on intellect tended to require less time to inhibit a prepotent motor response, and thus responded faster to presented visual cues. A possible explanation might be that a generally more pensive inclination characteristic of individuals scoring higher on intellect could mean they need less time for reflection (information processing) before they take action, which thus translates to a faster behavioral response to cues.

Prior research has linked impulsivity to various aspects of problem drinking (74–77). With an aim to better understand the underlying mechanism of such an association, we set out to identify which facets thereof are implicated with alcohol outcomes. In our sample, it was non-planning that turned out to be its most prevalent aspect, followed by motor and attentional impulsivity. Furthermore, our findings complement existing evidence on the links between personality contributors to impulsive behavior [e.g., (76, 78)]. The observed positive correlations between extraversion, attentional impulsivity and non-planning may reflect extraverted individuals’ elevated activity levels and excitement seeking nature, predisposing them to increased recklessness, lesser forethought and limited ability to focus on one item only. The negative association between agreeableness and non-planning may be accounted for by the thoughtfulness of others (and consequences of actions toward them), which is characteristic of highly agreeable persons and lacking in their impulsive counterparts. Likewise, negative associations between intellect, motor impulsivity and, again, non-planning illustrate the inverse relationship between the intellectual fondness of reflection and rash, reckless action with little precaution.

Against our expectations, no significant links were observed between self-report impulsivity and behavioral control. As such, this might be a relevant finding to support the notion that impaired control represents a related but separate construct rather than a facet of impulsivity, as postulated in other works (45). For impulsive behavior has been analyzed based on both impulsive self-reported personality traits *and* behavioral tasks [e.g., (79–81)], showing little overlap between the two (self-report and behavioral) measures and suggesting that they could actually assess distinct tendencies (79). This might illustrate how self-report measures could be construed as relative to the emotional/motivational underpinnings of impulsive behavior, while behavioral tasks reflect cognitive processes implicated in such behaviors, thus further supporting the distinct nature of the two. The interaction of both mechanisms is postulated to affect behavior, making their study highly encouraged for a comprehensive understanding of impulsive behavior in substance use disorders (80). This drinking-related reduced capacity of control may also be understood as a component of a more general breakdown in behavioral control related to alcohol use (48), and although linked to impulsivity, another complex, multi-faceted construct with relevance to various aspects of alcohol use disorders and other addictive behaviors (81–83), represent an independent theoretical entity. This means that in otherwise impulsive individuals, dysregulated response to alcohol might constitute a specific manifestation of general tendencies toward impaired behavioral control, but it may also occur in those who do not exhibit generally impulsive traits. Alternatively, the incongruous results of the two applied impulsivity measures suggesting considerable qualitative differences between them could be attributed to and imply deficits within metacognitive processes, i.e., little awareness of their actual behavioral impulsivity level and/or an unrealistic representation of their functioning in AUD patients c.f. (84). Such meta-cognitive bias could be another way to explain distinct self-report and experimental outcomes. This then goes to show that both impulsivity and impaired control in AUD cohorts, as well as the interplay between them remain all the more worthy of further theoretical and empirical attention.

Interestingly, depression symptomatology level proved to be related to all three aspects of self-report impulsivity (attentional, motor, and non-planning), in that more depressive participants tended to report poorer overall behavioral control, i.e., the levels of all facets thereof were higher in more depressive individuals. A more depressive mood therefore predicted a reduced ability to focus attention, the tendency to act without thinking and give little or no thought to consequences of own actions. We therefore found, as did other authors previously, that depression symptoms may interfere with drinking control, especially when coupled with tendencies to react impulsively to negative affect (49). Loss of behavioral control may occur in response to expectations that alcohol use could alleviate negative emotion, triggered by tendencies to act on impulses. This way drinking could be used as a means to regulate negative affect (85). Those suffering from persistent depressed mood and negative affective states could therefore prove more vulnerable to engage in coping-motivated drinking, expecting alcohol use to relieve tension and experiencing failures in control when responding impulsively to these expectations. Hence, symptoms of depression may contribute to increasing coping-motivated drinking and/or reducing regulatory capacities underlying adaptive coping.

Certain limitations of the current study outline promising research directions. Most notably, our findings were based on a rather small and homogenous (also gender-wise) sample of treatment-seeking rehabilitation center inpatients, which could contribute to a particular personality profile thereof. Limited variability in terms of Go/NoGo errors and depression symptomatology alongside little control for covariates mark significant study limitations, precluding meaningful conclusions concerning a prepotent response inhibition. Future research could therefore consider its replication in larger alcohol use populations with greater variability in demographics and drinking outcomes. Similarly, while the subjective facet of behavioral control seems to be well captured *via* the use of self-report tools, they tend to lack the objectivity offered by e.g., informant ratings or experimental paradigms. In an effort to reduce reliance on self-report measures, future research endeavors could be further extended to boost ecological validity and shift away from the conventional clinical settings toward the virtual reality-based trials. Yet another promising research direction neglected here but emerging from this study is the investigation of metacognitive ability in AUD patients with the likely neurobiological contributions to this phenomenon.

## Conclusion

In conclusion, the current study demonstrates the significant effect of conscientiousness, intellect, and depression symptomatology on impulsivity and processing speed, suggesting that personality and depression level may serve as important predictors of behavioral control measures in recovering drinkers. No significant links were observed between impulsivity and cognitive response inhibition suggesting that the two measures of behavioral control support a separate nature of the two notions. Therefore, our findings go above and beyond identifying the mere links of personality- and control-related factors with alcohol use outcomes, but rather they shed light on the interactions underlying the mechanisms implicated in the development and maintenance of AUDs and as such constitute significant evidence in alcohol addiction research.

## Data Availability Statement

The raw data supporting the conclusions of this article will be made available by the authors, without undue reservation.

## Ethics Statement

The studies involving human participants were reviewed and approved by the Bioethics Committee of the Pomeranian Medical University in Szczecin, Poland. The patients/participants provided their written informed consent to participate in this study.

## Author Contributions

ZL, AM, LM, SK, AN, JW, and JK-M contributed to the conception and design of the study. ZL, TS, AJ, ET, and JK-M organized the database. ZL and ET performed the statistical analysis. ZL wrote the first draft of the manuscript. All authors contributed to manuscript revision, read, and approved the submitted version.

## Conflict of Interest

The authors declare that the research was conducted in the absence of any commercial or financial relationships that could be construed as a potential conflict of interest.

## Publisher’s Note

All claims expressed in this article are solely those of the authors and do not necessarily represent those of their affiliated organizations, or those of the publisher, the editors and the reviewers. Any product that may be evaluated in this article, or claim that may be made by its manufacturer, is not guaranteed or endorsed by the publisher.

## References

[1] Worley J. Virtual reality for individuals with substance use disorders. *J Psychosoc Nurs Ment Health Serv.* (2019) 57:15–9. 10.3928/02793695-20190430-01 31162622

[2] World Health Organization. *World Health Statistics.* Geneva: World Health Organization (2018).

[3] Swift RM Aston ER. Pharmacotherapy for alcohol use disorder: current and emerging therapies. *Harv Rev Psychiatry.* (2015) 23:122–33. 10.1097/HRP.0000000000000079 25747925 PMC4790835

[4] Segawa T Baudry T Bourla A Blanc JV Peretti CS Mouchabac S Virtual reality (VR) in assessment and treatment of addictive disorders: a systematic review. *Front Neurosci.* (2020) 13:e1409. 10.3389/fnins.2019.01409 31998066 PMC6965009

[5] Hernández-Serrano O Ghiţă A Figueras-Puigderrajols N Fernández-Ruiz J Monras M Ortega L Predictors of changes in alcohol craving levels during a virtual reality cue exposure treatment among patients with alcohol use disorder. *J Clin Med.* (2020) 9:3018. 10.3390/jcm9093018 32962176 PMC7565669

[6] Bechara A. Decision making, impulse control and loss of willpower to resist drugs: a neurocognitive perspective. *Nat Neurosci.* (2005) 8:1458–63. 10.1038/nn1584 16251988

[7] World Health Organization. *The ICD-10 Classification of Mental and Behavioral Disorders: Clinical Descriptions and Diagnostic Guidelines.* Geneva: World Health Organization (1992).

[8] Paliwal P Hyman SM Sinha R. Craving predicts time to cocaine relapse: further validation of the now and brief versions of the cocaine craving questionnaire. *Drug Alcohol Depend.* (2008) 93:252–9. 10.1016/j.drugalcdep.2007.10.002 18063320 PMC2254317

[9] Galloway GP Singleton EG. Methamphetamine treatment project corporate authors. How long does craving predict use of methamphetamine? Assessment of use one to seven weeks after the assessment of craving. *Subst Abus.* (2008) 26:63–79. 10.4137/SART.S775 PMC2773437 19898674

[10] Saunders J Degenhardt L Reed G Poznyak V. Alcohol use disorders in ICD-11: past, present and future. *Alcohol Clin Exp Res.* (2015) 43:1617–31. 10.1111/acer.14128 31194891

[11] Castellanos-Ryan N Brière FN O’Leary-Barrett M Banaschewski T Bokde A Bromberg U The structure of psychopathology in adolescence and its common personality and cognitive correlates. *J Abnorm Psychol.* (2016) 125:1039–52. 27819466 10.1037/abn0000193 PMC5098414

[12] Ellingson JM Richmond-Rakerd LS Statham DJ Martin NG Slutske WS. Most of the genetic covariation between major depressive and alcohol use disorders is explained by trait measures of negative emotionality and behavioral control. *Psychol Med.* (2016) 46:2919–30. 10.1017/S0033291716001525 27460396 PMC9361478

[13] Malouff JM Thorsteinsson EB Rooke SE Schutte NS. Alcohol involvement and the five-factor model of personality: a meta-analysis. *J Drug Educ.* (2007) 37:277–94. 10.2190/DE.37.3.d 18047183

[14] Oreland L Lagravinese G Toffoletto S Nilsson KW Harro J Robert Cloninger C Personality as an intermediate phenotype for genetic dissection of alcohol use disorder. *J Neural Transm.* (2018) 125:107–30. 28054193 10.1007/s00702-016-1672-9 PMC5754455

[15] Lui PP Chmielewski M Trujillo M Morris J Pigott TD. Linking big five personality domains and facets to alcohol (Mis)use: a systematic review and meta-analysis. *Alcohol Alcohol.* (2022) 57:58–73. 10.1093/alcalc/agab030 33893471

[16] Newton NC Conrod PJ Slade T Carragher N Champion KE Barrett EL The long-term effectiveness of a selective, personality-targeted prevention program in reducing alcohol use and related harms: a cluster randomized controlled trial. *J Child Psychol Psychiatry.* (2016) 57:1056–65. 10.1111/jcpp.12558 27090500

[17] O’Leary-Barrett M Castellanos-Ryan N Pihl RO Conrod PJ. Mechanisms of personality-targeted intervention effects on adolescent alcohol misuse, internalizing and externalizing symptoms. *J Consult Clin Psychol.* (2016) 84:438–52. 10.1037/ccp0000082 26881449

[18] Hakulinen C Elovainio M Batty GD Virtanen M Kivimäki M Jokela M. Personality and alcohol consumption: pooled analysis of 72,949 adults from eight cohort studies. *Drug Alcohol Depend.* (2015) 151:110–4. 10.1016/j.drugalcdep.2015.03.008 25823906 PMC4447572

[19] Liu S Wang M Zhan Y Shi J. Daily work stress and alcohol use: testing the cross-level moderation effects of neuroticism and job involvement. *Pers Psychol.* (2009) 62:575–97.

[20] Livingston NA Oost KM Heck NC Cochran BN. The role of personality in predicting drug and alcohol use among sexual minorities. *Psychol Addict Behav.* (2015) 29:414–9. 10.1037/adb0000034 25347022 PMC4411203

[21] Mushquash CJ Stewart SH Mushquash AR Comeau MN McGrath PJ. Personality traits and drinking motives predict alcohol misuse among Canadian aboriginal youth. *Int J Ment Health Addict.* (2014) 12:270–82.

[22] Raynor DA Levine H. Associations between the five-factor model of personality and health behaviors among college students. *J Am Coll Health.* (2009) 58:73–81. 19592356 10.3200/JACH.58.1.73-82

[23] Cooper ML Kuntsche E Levitt A Barber LL Wolf S. Motivational models of substance use a review of theory and research on motives for using alcohol, marijuana, and tobacco. In: Sher KJ editor. *The Oxford Handbook of Substance use and Substance use Disorders.* Oxford: Oxford University Press (2015). 10.2190/Q3YY-M40L-H4A2-8404

[24] Kuntsche E Knibbe R Gmel G Engels R. Who drinks and why? A review of socio-demographic, personality, and contextual issues behind the drinking motives in young people. *Addict Behav.* (2006) 31:1844–57. 10.1016/j.addbeh.2005.12.028 16460883

[25] Chinneck A Thompson K Dobson KS Stuart H Teehan M Stewart SH. Neurotic personality traits and risk for adverse alcohol outcomes: chained mediation through emotional disorder symptoms and drinking to cope. *Subst Use Misuse.* (2018) 53:1730–41. 10.1080/10826084.2018.1432647 29393722

[26] Armeli S Carney MA Tennen H Affleck G O’Neil TP. Stress and alcohol use: a daily process examination of the stressor–vulnerability model. *J Pers Soc Psychol.* (2000) 78:979–94. 10.1037//0022-3514.78.5.979 10821203

[27] Ebbert AM Patock−Peckham JA Luk JW Voorhies K Warner O Leeman RF. The mediating role of anxiety sensitivity in uncontrolled drinking: a look at gender−specific parental influences. *Alcohol Clin Exp Res.* (2018) 42:914–25. 10.1111/acer.13631 29573434 PMC5915872

[28] Lee MR Ellingson JM Sher KJ. Integrating social−contextual and intrapersonal mechanisms of ‘maturing out’: joint influences of familial−role transitions and personality maturation on problem−drinking reductions. *Alcohol Clin Exp Res.* (2015) 39:1775–87. 10.1111/acer.12816 26247314 PMC4558380

[29] Zilberman N Yadid G Efrati Y Neumark Y Rassovsky Y. Personality profiles of substance and behavioral addictions. *Addict Behav.* (2018) 82:174–81. 10.1016/j.addbeh.2018.03.007 29547799

[30] Zilberman N Yadid G Efrati Y Rassovsky Y. Who becomes addicted and to what? psychosocial predictors of substance and behavioral addictive disorders. *Psychiatry Res.* (2020) 291:113221. 10.1016/j.psychres.2020.113221 32562935

[31] Ernst LH Plichta MM Dresler T Zesewitz AK Tupak SV Haeussinger FB Prefrontal correlates of approach preferences for alcohol stimuli in alcohol dependence. *Addict Biol.* (2014) 19:497–508. 10.1111/adb.12005 23145772

[32] Winstanley CA Olausson P Taylor JR Jentsch JD. Insight into the relationship between impulsivity and substance abuse from studies using animal models. *Alcohol Clin Exp Res.* (2010) 34:1306–18. 10.1111/j.1530-0277.2010.01215.x 20491734 PMC3380443

[33] Haeny AM Gueorguieva R Morean ME Krishnan-Sarin S DeMartini KS Pearlson GD The association of impulsivity and family history of alcohol use disorder on alcohol use and consequences. *Alcohol Clin Exp Res.* (2020) 44:159–67. 31693193 10.1111/acer.14230 PMC6981005

[34] Liu W Chen XJ Wen YT Winkler MH Paul P He YL Memory retrieval-extinction combined with virtual reality reducing drug craving for methamphetamine: study protocol for a randomized controlled trial. *Front Psychiatry.* (2020) 11:e322. 10.3389/fpsyt.2020.00322 32411025 PMC7202246

[35] Jakubczyk A Trucco EM Kopera M Kobyliński P Suszek H Fudalej S The association between impulsivity, emotion regulation, and symptoms of alcohol use disorder. *J Subst Abuse Treat.* (2018) 91:49–56. 10.1016/j.jsat.2018.05.004 29910014 PMC6020846

[36] Khemiri L Franck J Jayaram-Lindström N. Effect of alcohol use disorder family history on cognitive function. *Psychol Med.* (2020) 52:757–69. 10.1017/S003329172000238X 32662376

[37] Tomassini A Struglia F Spaziani D Pacifico R Stratta P Rossi A. Decision making, impulsivity, and personality traits in alcohol-dependent subjects. *Am J Addict.* (2012) 21:263–7. 10.1111/j.1521-0391.2012.00225.x 22494229

[38] Papachristou H Nederkoorn C Corstjens J Jansen A. The role of impulsivity and perceived availability on cue-elicited craving for alcohol in social drinkers. *Psychopharmacology (Berl).* (2012) 224:145–53. 10.1007/s00213-012-2747-4 22638812 PMC3465646

[39] Ferguson SG Shiffman S. The relevance and treatment of cue-induced cravings in tobacco dependence. *J Subst Abus Treat.* (2009) 36:235–43. 10.1016/j.jsat.2008.06.005 18715743

[40] Brody AL Mandelkern MA London ED Childress AR Lee GS Bota RG Brain metabolic changes during cigarette craving. *Arch Gen Psychiatry.* (2002) 59:1162–72. 10.1001/archpsyc.59.12.1162 12470133

[41] Franklin TR Wang J Sciortino N Harper D Li Y Ehrman R Limbic activation to cigarette smoking cues independent of nicotine withdrawal: a perfusion fMRI study. *Neuropsychopharmacology.* (2007) 32:2301–9. 10.1038/sj.npp.1301371 17375140

[42] McClernon FJ Kozink RV Lutz AM Rose JE. 24-h smoking abstinence potentiates fMRI-BOLD activation to smoking cues in cerebral cortex and dorsal striatum. *Psychopharmacology.* (2009) 204:25–35. 10.1007/s00213-008-1436-9 19107465 PMC2810714

[43] Heather N Tebbutt JS Mattick RP Zamir R. Development of a scale for measuring impaired control over alcohol consumption: a preliminary report. *J Stud Alcohol.* (1993) 54:700–9. 10.15288/jsa.1993.54.700 8271806

[44] Leeman RF Toll BA Taylor LA Volpicelli JR. Alcohol-induced disinhibition expectancies and impaired control as prospective predictors of problem drinking in undergraduates. *Psychol Addict Behav.* (2009) 23:553–63. 10.1037/a0017129 20025361 PMC2805107

[45] Leeman RF Patock-Peckham JA Potenza MN. Impaired control over alcohol use: an under-addressed risk factor for problem drinking in young adults? *Exp Clin Psychopharmacol.* (2012) 20:92–106. 10.1037/a0026463 22182417 PMC3613490

[46] Leeman RF Beseler CL Helms CM Patock−Peckham JA Wakeling VA Kahler CW. A brief, critical review of research on impaired control over alcohol use and suggestions for future studies. *Alcohol Clin Exp Res.* (2014) 38:301–8. 10.1111/acer.12269 24117468 PMC3946792

[47] Vaughan CL Stangl BL Schwandt ML Corey KM Hendershot CS Ramchandani VA. The relationship between impaired control, impulsivity, and alcohol self-administration in nondependent drinkers. *Exp Clin Psychopharmacol.* (2019) 27:236–46. 10.1037/pha0000247 30688502 PMC6776085

[48] Patock-Peckham JA Cheong J Balhorn ME Nagoshi CT. A social learning perspective: a model of parenting styles, self-regulation, perceived drinking control, and alcohol use and problems. *Alcohol Clin Exp Res.* (2001) 25:1284–92. 11584147

[49] Zaso MJ Hendershot CS Wardell JD Bagby RM Pollock BG Quilty LC. Characterizing the role of impaired control over alcohol in associations of impulsive personality traits with alcohol use as a function of depressive disorder. *Addict Behav.* (2021) 112:106633. 10.1016/j.addbeh.2020.106633 32949836 PMC8034598

[50] Boden JM Fergusson DM. Alcohol and depression. *Addiction.* (2011) 106:906–14. 21382111 10.1111/j.1360-0443.2010.03351.x

[51] Institute of Health Metrics and Evaluation. *Global Health Data Exchange (GHDx).* (2022). Available online at: http://ghdx.healthdata.org/gbd-results-tool?params=gbd-api-2019-permalink/d780dffbe8a381b25e1416884959e88b (accessed January 23, 2022)

[52] Hasin DS Sarvet AL Meyers JL Saha TD Ruan WJ Stohl M Epidemiology of adult DSM-5 major depressive disorder and its specifiers in the United States. *JAMA Psychiatry.* (2018) 75:336–46. 29450462 10.1001/jamapsychiatry.2017.4602 PMC5875313

[53] Koob GF Volkow ND. Neurocircuitry of addiction. *Neuropsychopharmacology.* (2010) 35:217–38. 10.1038/npp.2009.110 19710631 PMC2805560

[54] Covid-19 Mental Disorders Collaborators. Global prevalence and burden of depressive and anxiety disorders in 204 countries and territories in 2020 due to the COVID-19 pandemic. *Lancet.* (2021) 398:1700–12. 10.1016/S0140-6736(21)02143-7 34634250 PMC8500697

[55] Mellentin AI Nielsen AS Ascone L Wirtz J Samochowiec J Kucharska-Mazur J A randomized controlled trial of a virtual reality based, approach-avoidance training program for alcohol use disorder: a study protocol. *BMC Psychiatry.* (2020) 20:340. 10.1186/s12888-020-02739-1 32605614 PMC7324964

[56] Sheehan DV Lecrubier Y Sheehan KH Amorim P Janavs J Weiller E The mini-international neuropsychiatric interview (MINI): the development and validation of a structured diagnostic psychiatric interview for DSM-IV and ICD-10. *J Clin Psychiatry.* (1998) 59 (Suppl. 20):22–33;quiz34–57. 9881538

[57] Sheehan DV. *Mini International Neuropsychiatric Interview 7.0.* Jacksonville, FL: Medical Outcomes Systems (2015).

[58] Goldberg LR. A broad-bandwidth, public-domain, personality inventory measuring the lower-level facets of several five-factor models. In: Mervielde I Deary I De Fruyt F Ostendorf F editors. *Personality Psychology in Europe.* (Vol. 7), Tilburg: Tilburg University Press (1999). p. 7–28.

[59] Costa PT Jr. McCrae RR. *Revised NEO Personality Inventory (NEO-PI-R) and NEO Five-Factor Inventory (NEO-FFI) professional manual.* Odessa, FL: Psychological Assessment Resources (1992).

[60] Beck AT Steer RA Ball R Ranieri W. Comparison of beck depression inventories -IA and -II in psychiatric outpatients. *J Pers Assess.* (1996) 67:588–97. 10.1207/s15327752jpa6703_13 8991972

[61] Patton JH Stanford MS Barratt ES. Factor structure of the barratt impulsiveness scale. *J Clin Psychol.* (1995) 51:768–74. 8778124 10.1002/1097-4679(199511)51:6<768::aid-jclp2270510607>3.0.co;2-1

[62] Gordon B Caramazza A. Lexical decision for open-and closed-class words: failure to replicate differential frequency sensitivity. *Brain Lang.* (1982) 15:143–60. 10.1016/0093-934x(82)90053-0 6184120

[63] Gordon B. Lexical access and lexical decision: mechanisms of frequency sensitivity. *J Verbal Learn Verbal Behav.* (1983) 22:24–44.

[64] Meyers LS Gamst GC Guarino AJ. *Performing Data Analysis using IBM SPSS.* Hoboken, NJ: John Wiley & Sons (2013).

[65] Raykov T. On the use of confirmatory factor analysis in personality research. *Pers Individ Differ.* (1998) 24:291–3. 10.1016/S0191-8869(97)00159-1

[66] Joreskog KG Sorbom D. *LISREL VI: Analysis of Linear Structural Relationships by Maximum Likelihood, Instrumental Variables, and Least Squares Methods.* Mooresville, IN: Scientific Software (1986).

[67] Bentler PM. Comparative fit indexes in structural models. *Psychol Bull.* (1990) 107:238–46. 10.1037/0033-2909.107.2.238 2320703

[68] Hu L Bentler PM. Cutoff criteria for fit indexes in covariance structure analysis: conventional criteria versus new alternatives. *Struct Equ Modeling.* (1999) 66:1–55.

[69] Kotyuk E Farkas J Magi A Eisinger A Király O Vereczkei A The psychological and genetic factors of the addictive behaviors (PGA) study. *Int J Methods Psychiatr Res.* (2019) 28:e1748. 10.1002/mpr.1748 30402898 PMC6877275

[70] MacKillop J Weafer J C Gray J Oshri A Palmer A de Wit H. The latent structure of impulsivity: impulsive choice, impulsive action, and impulsive personality traits. *Psychopharmacology (Berl).* (2016) 233:3361–70. 10.1007/s00213-016-4372-0 27449350 PMC5204128

[71] Minhas M Murphy CM Balodis IM Acuff SF Buscemi J Murphy JG Multidimensional elements of impulsivity as shared and unique risk factors for food addiction and alcohol misuse. *Appetite.* (2021) 159:105052. 10.1016/j.appet.2020.105052 33309712

[72] Smith JL Mattick RP Jamadar SD Iredale JM. Deficits in behavioral inhibition in substance abuse and addiction: a meta-analysis. *Drug Alcohol Depend.* (2014) 145:1–33. 10.1016/j.drugalcdep.2014.08.009 25195081

[73] Sjoerds Z Van Den Brink W Beekman ATF Penninx BWJH Veltman DJ. Response inhibition in alcohol-dependent patients and patients with depression/anxiety: a functional magnetic resonance imaging study. *Psychol Med.* (2014) 44:1713–25. 10.1017/S0033291713002274 24016382

[74] Herman AM Duka T. Facets of impulsivity and alcohol use: what role do emotions play? *Neurosci Biobehav Rev.* (2019) 106:202–16. 10.1016/j.neubiorev.2018.08.011 30343823

[75] Macpherson L Magidson JF Reynolds EK Kahler CW Lejuez CW. Changes in sensation seeking and risk-taking propensity predict increases in alcohol use among early adolescents. *Alcohol Clin Res.* (2010) 34:1400–8. 10.1111/j.1530-0277.2010.01223.x 20491737 PMC3123723

[76] Stautz K Cooper A. Impulsive behavior-related personality traits and adolescent alcohol use: a meta-analytic review. *Clin Psychol Rev.* (2013) 33:574–92. 10.1016/j.cpr.2013.03.003 23563081

[77] Tarter RE Hegedus AM Gavaler JS. Hyperactivity in sons of alcoholics. *J Stud Alcohol.* (1985) 46:259–61. 4010305 10.15288/jsa.1985.46.259

[78] Birkley EL Smith GT. Recent advances in understanding the personality underpinnings of impulsive behavior and their role in risk for addictive behaviors. *Curr Drug Abuse Rev.* (2011) 4:215–27. 10.2174/1874473711104040215 22126707 PMC3954823

[79] Cyders MA Coskunpinar A. Measurement of constructs using self-report and behavioral lab tasks: is there overlap in nomothetic span and construct representation for impulsive behavior? *Clin Psychol Rev.* (2011) 31:965–82. 10.1016/j.cpr.2011.06.001 21733491

[80] Sharma L Markon KE Clark LA. Toward a theory of distinct types of”impulsive” behaviors: a meta-analysis of self-report and behavioral measures. *Psychol Bull.* (2014) 140:374–408. 10.1037/a0034418 24099400

[81] Verdejo-García A Lawrence AJ Clark L. Impulsive behavior as a vulnerability marker for substance-use disorders: review of findings from high-risk research, problem gamblers and genetic association studies. *Neurosci Biobehav Rev.* (2008) 32:777–810. 10.1016/j.neubiorev.2007.11.003 18295884

[82] Dick DM Smith GT Olausson P Mitchell S Leeman RF O’Malley SS Understanding the construct of impulsivity and its relationship to alcohol use disorders. *Addict Biol.* (2010) 15:217–26. 20148781 10.1111/j.1369-1600.2009.00190.x PMC2895996

[83] Whiteside SP Lynam DR. The five factor model and impulsivity: using a structural model of personality to understand impulsivity. *Pers Ind Differ.* (2001) 30:669–89.

[84] Balconi M Finocchiaro R Campanella S. Reward sensitivity, decisional bias, and metacognitive deficits in cocaine drug addiction. *J Addict Med.* (2014) 8:399–406. 10.1097/ADM.0000000000000065 25303980

[85] Greeley J Oei T. Alcohol and tension reduction. In: Leonard KE Blane HT editors. *The Guilford Substance Abuse Series: Psychological Theories of Drinking and Alcoholism.* New York, NY: The Guilford Press (1999). p. 14–53.

